# Editorial: Aging and Mental Health

**DOI:** 10.3389/fnagi.2017.00025

**Published:** 2017-02-10

**Authors:** Lia Fernandes, Constança Paúl

**Affiliations:** ^1^Faculty of Medicine, University of Porto, Center for Health Technology and Services Research (CINTESIS)Porto, Portugal; ^2^Research and Education Unit on Ageing (UNIFAI), Institute of Biomedical Sciences Abel Salazar, University of PortoPorto, Portugal

**Keywords:** aging, mental health, dementia, depression, delirium, chronic diseases, frailty, disability

According to the “Mental Health Action Plan 2013–2020” (WHO, 2013), mental health is an integral part of health and well-being and includes not only individual characteristics but also social, cultural, economic political, and environmental factors. The report recognizes that depending on the context certain groups, such as older people, are at higher risk of having mental health problems and consequently having higher rates of disability and mortality.

The main issues concerning aging and mental health are prevention, early diagnosis, recognition of major diseases, treatment and quality of life interventions, at both individual and community level.

Available knowledge about the aging process and mental health is still insufficient and the challenges of aging populations claim more research efforts into clinical conditions, older people's needs, and pathways of care. With the increase in average life expectancy, chronic conditions inherent to aging, such as dementias (in particular Alzheimer's Disease), are inevitably growing along with related behavioral and psychological disorders, which highlights the need for specific interventions in elderly mental health problems. Beside dementia and mild cognitive impairment, other issues like frailty, delirium and the risk for mental health problems or the unmet needs of older people, require substantially more attention from professionals and policy agents. The burden of mental health problems is frequently considered as an inevitable part of the process of aging, worsening the already negative stereotype about being old. Mental health issues, particularly those affecting old age are frequently underestimated, adding to the suffering of a large number of people who could be treated and benefit from diverse social and health care interventions to enhance their well-being. The comprehension of mechanisms underlying diseases, on a time diagnosis, and customized interventions will be much more cost-effective than just allowing the disease to progress, leading to the institutionalization of individuals, which has proved to be an adverse and expensive outcome, both for individuals and the community.

Taking this into consideration, the present Research Topic on “Aging and Mental Health” by Frontiers in Aging Neuroscience makes a contribution with updates and different perspectives on this important theme, developed over 17 papers. These updates focus on cognitive functions, neural and cellular mechanisms, delirium and mild cognitive impairment as well as the effects of exercise and health care settings.

The authors Lim and Yu have conducted an interesting review on the relation between aging and wisdom: age-related changes in economic and social decision making. They discuss this topic based on a model proposing five subcomponents of wisdom: (1) prosocial behavior in experimental economic games and competitive situations; (2) resolving social conflicts; (3) emotional homeostasis; (4) self-reflection; (5) dealing effectively with uncertainty in the domains of risk, ambiguity and intertemporal choice. They highlight that older adults outperform young adults in certain subcomponents of wisdom, but the exact relationship between old age and each subcomponent remains unclear.

Alves et al. addresses an interesting topic, based on the diversion paradigm developed by Delaney et al. (2010). The authors aim to determine whether thinking about an autobiographical memory interferes with the recall of recently encoded information, and to explore the degree of forgetfulness depending on the temporal distance from the diversionary thought. They conclude that the expected amnesic effect of diversionary thought was reached, but the temporal distance (old event vs. recent event) did not influence the recall of previously encoded information.

Szatloczki et al. point out the role of linguistic screening for early detection of Alzheimer's disease. The authors have produced a comprehensive review, concluding that AD can be more sensitively detected with the help of linguistic analysis than with other cognitive examinations, which would have relevant clinical implications.

The paper by Meng et al. examines the impact of aging on the brain's susceptibility to affective pictures of varying emotional intensities, with results that suggest that older adults are more resistant to the impact of negative stimuli, while they are equipped with enhanced attentional bias for positive stimuli.

Two studies about delirium are presented in this Research Topic. The first one by Androsova et al. is an interesting, clinically relevant literature review focused on the “Biomarkers of postoperative delirium and cognitive dysfunction”. Bearing in mind that the pathogenesis of these postoperative cognitive impairments is multifactorial, the authors emphasize that the application of integrated systems biology has the potential to reconstruct the underlying network of molecular mechanisms and help in the identification of prognostic and diagnostic biomarkers. The second one, produced by Shi et al., finds a good reliability of MDAS and the cutoff point for diagnosis of delirium.

Coelho et al. have written an article about “Determinants of frailty: the added value of assessing medication”. With this they analyse in general the determinants that predict frailty, as well as each frailty domain (physical, psychological, and social), considering the integral conceptual model of frailty. Particularly, the contribution of different daily-consumed medication is analyzed. The results add important information about which factors may precipitate states of high vulnerability in community dwelling elderly.

The article “Mismatch negativity (MMN) latency as a biomarker of amnestic mild cognitive impairment in Chinese rural elders” by Ji et al., aims to assess the mismatch negativity (MMN) component, a correlate of the automatic detection of changes in the acoustic environment, in healthy adults, and adults with amnestic mild cognitive impairment (aMCI). They find that while no difference was observed in amplitude between the two groups, there was a significant increase in the latency of the MMN in the aMCI group, which could be a sensitive, specific biomarker.

Akintola et al. have written a broad systematic review and meta-analysis concerning “Subclinical hypothyroidism (SCH) and cognitive function in people over 60 years”. This meta-analysis, performed to assess available evidence on the association of SCH with cognition in community dwelling, relatively healthy older adults, found no evidence concerning this association.

Bearing in mind the well-known importance of regular exercise in benefiting mental and physical health in the elderly Santa Mina et al. have written the article “The acute effects of exercise on cortical excitation and psychosocial outcomes in men treated for prostate cancer: a randomized controlled trial”. They have evaluated and presented novel findings of the effect of a single bout of exercise (low-moderate intensity) on psychological well-being and cortical silent period in prostate cancer survivors.

Paúl et al. have conducted a cross-sectional study about “Perceived risk of mental health problems in primary care” using the Portuguese version of RISC as a useful tool for early identification of mental health concerns in older patients. This study emphasizes the importance of healthcare professionals identifying patients at high risk of adverse outcomes early on in order to direct an appropriate intervention.

Bearing in mind that unmet needs are becoming acknowledged as better predictors of the worst prognostic outcomes than common measures of functional or cognitive decline, the article “Needs in nursing homes and their relation with cognitive and functional decline, behavioral and psychological symptoms” by Ferreira et al. will be a valuable aid to all those interested in learning about this issue. The authors have found more unmet needs associated with the worst outcomes, demonstrating that the needs of those institutionalized elderly remain under-diagnosed and untreated.

In the same setting of the nursing home, Caravau and Martin have written the paper “Direct costs of dementia in nursing homes”. The authors deal with an interesting and poorly-studied topic, concluding that direct costs of dementia patients in Portugal exceed the costs of similar non-demented patients by a significant degree, which is in agreement with previous European studies, with direct implications for the provision of services for the management of the increasing prevalence of dementia.

The manuscript by Bastos et al. analyses “The importance of neighborhood ecological assets in community-dwelling old people's aging outcomes: A study in Northern Portugal,” concludes that cognitive performance decreases in persons with the worst outdoor mobility, and also that depressive symptoms are less common with a greater number of recreation opportunities, suggesting that aging policies and practices must be ecologically embedded.

Finally, two commentaries have been written, the first of which also has a response. Craig has written a commentary on “Mental distress in patients with cerebral visual injury assessed with the German Brief Symptom Inventory” by Gall et al.. The second commentary written by Heine is about the article “Mental health and dual sensory loss (DSL) in older adults: a systematic review”, by Heine and Browning (2014). This text focuses on the lack of solid data on the present subject, and also the need to diagnose and manage DSL early and effectively.

We hope that this Frontiers Research Topic will be an enrichment for Ageing and Mental Health issues, with the efforts and commitments of all authors to whom we give our acknowledgement, as well as to the reviewers who have contributed by improving and clarifying these diverse contributions.

Finally, a special thanks for the support for publishing by the sponsor Pfizer, for three articles (Paúl et al.; Caravau and Martin; Bastos et al.). The sponsor did not play any role in the design, methods, data collection and analyses, or in the preparation of these articles.

## Author contributions

LF and CP have written this editorial for the Research Topic they have edited.

### Conflict of interest statement

The authors declare that the research was conducted in the absence of any commercial or financial relationships that could be construed as a potential conflict of interest.
